# Epigenetic signatures of local adaptation: differential expression of long non-coding RNAs in reciprocally transplanted *Mytilus chilensis*

**DOI:** 10.3389/fimmu.2026.1725909

**Published:** 2026-05-20

**Authors:** Marco Yévenes, Gajardo Gonzalo, Cristian Gallardo-Escárate

**Affiliations:** 1 Laboratorio de Genética, Acuicultura & Biodiversidad, Departamento de Ciencias Biológicas y Biodiversidad, Universidad de Los Lagos, Osorno, Chile; 2 Interdisciplinary Center for Aquaculture Research (INCAR), Universidad de Concepción, Concepción, Chile

**Keywords:** differential gene expression, epigenetics, lncRNAs, local adaptation, *Mytilus chilensis*

## Abstract

Genetic variation alone does not fully explain how marine organisms adapt locally; epigenetic mechanisms such as long non-coding RNAs (lncRNAs) may also contribute. This study explores expression differences associated with differentially expressed lncRNAs (DE-lncRNAs) in *Mytilus chilensis* from two environmentally contrasting natural seedbeds in the inner sea of Chiloé, Cochamó (41°S) and Yaldad (43°S), through a 91-day reciprocal transplant experiment. Twelve RNA-seq libraries were analyzed using 43011 sequences from the *M. chilensis* lncRNA database and the chromosome-level whole-genome sequence as references. Differential expression was assessed under the local vs. foreign (δ_LF_) and home vs. away (δ_HA_) criteria to evaluate lncRNA expression patterns consistent with local adaptation, using progressive significance thresholds (fold change ≥|3–10|, FDR p-value ≤0.05). Marked environmental differences between the two sites, combined with contrasting lncRNA expression profiles in self- and cross-transplanted mussels, highlight the potential contribution of epigenetic regulation to genomic patterns associated with local adaptation. Mussels from Yaldad exhibited more transcripts with extreme fold changes, consistent with greater sensitivity to translocation. Genome mapping linked 183 DE-lncRNAs with nearby protein-coding genes (NPC-genes), mainly lincRNAs, with predicted subcellular localization in cytoplasmic (50.2%) and nuclear (29.5%) compartments. Only five NPC-genes overlapped with previously identified candidate adaptive differentially expressed genes (caDEGs), two of which were associated with immune function and cellular recognition. These findings suggest that lncRNAs may represent a complementary epigenetic layer associated with adaptive responses of *M. chilensis* under heterogeneous environments and aquaculture-related translocations. This study provides novel insights into the interplay between genetic and epigenetic variation, laying a foundation for future research on the role of lncRNAs in adaptation, conservation, and sustainable resource management.

## 1 Introduction

Traditional genetics and evolutionary theory hold that adaptive phenotypes are shaped by natural selection acting on genetic variation caused by random mutations in DNA sequences (1). However, an emerging body of evidence indicates that genetic variation alone is insufficient to explain the diversity of ecologically relevant traits. This variation also depends on epigenetic mechanisms such as DNA methylation, chromatin modifications, and non-coding RNAs (2–4). These environmentally-induced epigenetic changes can be inherited (5, 6), providing a rapid and complementary inheritance system under changing environmental conditions (7, 8). Within this epigenetic landscape, long non-coding RNA (lncRNA) loci increasingly emerge as regulatory genes involved in chromatin organization, enhancer-like activity, and transcriptional condensates, with functions dependent on locus context and subcellular localization (9–11).

Long non-coding RNAs (lncRNAs) are transcripts longer than 200 nucleotides that, although transcribed similarly to mRNAs, lack conserved protein-coding domains (12, 13). Their expression is often highly tissue-specific and regulated across both spatial and temporal dimensions (14, 15). They are classified based on their genomic context relative to protein-coding genes (e.g., intergenic, antisense, or promoter-associated) and their biogenesis (16–18). Functionally, they are versatile regulators that can modulate gene expression through chromatin remodeling, transcriptional interference, and the recruitment of transcription factors, impacting both nearby genes (in cis) and distant genes (in trans) (19–21). An example of their complex regulatory roles is observed in the pearl oyster *Pinctada fucata*, where lncIRF-2 positively regulates the Interleukin-17 gene while simultaneously repressing its neighboring PfIRF-2 gene (22).

Assigning specific functions to individual lncRNAs remains a significant challenge due to their involvement within complex epigenetic networks and their additive effects on both local and distal gene regulation (19, 23). Despite this, a growing body of evidence shows that lncRNA expression is responsive to immune stimuli across a broad phylogenetic range, including terrestrial organisms (24, 25), marine vertebrates (26–28) and invertebrates (29–32). In marine mollusks, lncRNAs are differentially expressed under various natural and experimental conditions and are implicated in critical biological processes such as shell biomineralization, development, and immune defense (32–35). Evidence for a direct regulatory role is robust from studies on *Mytilus galloprovincialis*, where immune challenges lead to the coordinated differential expression of lncRNAs and their neighboring protein-coding genes (NPC-genes), indicating a cis-regulatory function (31, 36). Yet, the role of lncRNAs in local adaptation remains largely unexplored, particularly within an explicit ecological and evolutionary framework. Although previous studies in marine bivalves have described lncRNA responses to environmental stress (31, 32), these have not been directly evaluated in the context of local adaptation using reciprocal transplant experiments. In reciprocal transplant frameworks, local adaptation is commonly assessed using fitness contrasts such as local versus foreign (δ_LF_) and home versus away (δ_HA_) individuals, which capture complementary aspects of genotype performance across environmental context. Therefore, this study addresses this gap by investigating adaptive epigenomic variation mediated by differentially expressed lncRNAs in *Mytilus chilensis*, integrating transcriptomic data within a local adaptation framework.

*Mytilus chilensis* (Hupé, 1854) is an endemic blue mussel of great socio-ecological and economic significance in southern Chile due to its extensive use in aquaculture, making it a relevant model for studying epigenetic variation in adaptation to heterogeneous environments. This species is closely related to the northern hemisphere *M. edulis* species complex (37, 38) and inhabits rocky substrates in intertidal and subtidal zones along the South Pacific coast, from latitude 38°S (Bío Bío) to 53°S (Magallanes) (39). It experiences dynamic marine fluctuations of temperature, salinity, dissolved oxygen, and experience desiccation and ultraviolet radiation during tide fluctuation (40, 41). As an ecosystem engineer (42, 43) plays a key role in regulating phytoplankton flow, nutrient cycling, and remineralization of organic deposits in sediments. It has been subject of numerous ecological (44), eco-physiological (45), and adaptive genomic studies (34, 46–49). On the practical side, this species supports a world-class farming industry (50) centered in the inner sea of Chiloé Island (between latitudes 41,5°S and 43,5°S), southern Chile. This industry depends on the availability of juvenile individuals (seeds), which are collected from natural seedbeds and transferred to ecologically heterogeneous bays until harvest. Due to ongoing extraction and limited natural recruitment, some of these natural seedbeds have decreased in size and exhibited high levels of inbreeding (51). Cochamó and Yaldad are two natural seedbeds supporting the industry (52, 53), located in the northern and southern zones of the inner sea of Chiloé Island, respectively. Both locations are roughly 250 km apart and are separated by gradients in seawater temperature, currents, salinity, and Chlorophyll-a concentration from north to south (54–56). Consistent with these environmental contrasts, recent multi-tissue work in oysters shows salinity-driven, tissue-specific lncRNA responses and lncRNA–mRNA co-regulation, supporting the idea of environmentally responsive lncRNA regulation in marine bivalves (57).

As a gonochoric species, *M. chilensis* undergoes an annual gametogenic cycle, reaching sexual maturity in spring-summer. After fertilization, the planktonic larvae can drift in the water column for 20 to 45 days before settling, potentially traveling up to 30 km depending on oceanographic conditions (58–60). This dispersal capacity has been used to explain the low but significant genetic divergence and population structuring in this species, often interpreted as high genetic connectivity. For example, a microsatellite-based study have suggested that mussels from southern Chile form a single reproductive unit, with some regional exceptions (61). Genomic studies using SNPs and outlier SNPs have reported low overall genetic divergence, but with contrasting interpretations regarding the strength of local adaptation (52, 62). In line with the possibility of local adaptive differentiation, recent genomic evidence suggests that individuals of this species retain local adaptations in gene expression (49).

Using a reciprocal transplant experiment and outlier SNPs genome mapping, the study applied two operational criteria to assess patterns consistent with local adaptations (63, 64), capturing complementary dimensions of genotype–environment interactions: i) comparing gene expression related to fitness traits in local versus immigrant individuals (local vs. foreign criterion, δ_LF_) and ii) assessing gene expression in native versus non-native environments (home vs. away criterion, δ_HA_). The study identified differentially expressed candidate adaptive genes (caDEGs) associated to key physiological processes, which showed significant differential expression in individuals from two ecologically contrasting seedbeds (Cochamó and Yaldad). Additionally, outlier SNPs distributed across the genome were associated to adaptive candidate genes involved in osmoregulation, oxidative stress, and oxygen management. These findings highlight the role of local environments in shaping adaptive genomic response of *M. chilensis* and point to the complexity of evolutionary processes, with potential implications for conservation and management of the species.

Given the importance of epigenetic variation for evolutionary change and adaptation to heterogeneous environments (4, 7, 8), this study hypothesizes that the complex environmental differences experienced by natural seedbeds of the Chilean blue mussel within the inner sea of Chiloé Island lead to differentially expressed lncRNAs (DE-lncRNAs) in their genome, associated with neighboring caDEGs in the *M. chilensis* genome. Such epigenetic differentiation may contribute to patterns consistent with local adaptation in gene expression observed in individuals inhabiting ecologically distinct seedbeds. Therefore, this study aims to identify differentially expressed long non-coding RNAs (DE-lncRNAs) and explore how their expression relates to previously reported candidate adaptive differentially expressed genes (caDEGs).

To test this hypothesis, previously reported transcriptomic data from a 91-day reciprocal transplant experiment (49), conducted between two ecologically contrasting seedbeds, were used to assess lncRNA differential expression in control (self-transplanted) and experimental (cross-transplanted) individuals under the δ_LF_ and δ_HA_ criteria. Together, these analyses provide a framework to examine how epigenetic and genetic variation may interact in population-level responses to environmental change and habitat translocation.

## 2 Materials and methods

### 2.1 Study sites and sampling

Raw oceanographic data, including temperature (°C), currents (m/s), salinity (psu), and seawater age (days) as an estimate of dissolved oxygen (65), were obtained from the CHONOS database (http://chonos.ifop.cl/), managed by the Chilean Institute of Fisheries Enhancement (IFOP). Data were collected for Cochamó, located at the northernmost tip of the inner sea of Chiloé Island (41°28’23.77’’ S, 72°18’38.61’’ W), an estuarine bay with a constant influx of freshwater; and Yaldad, at the southernmost tip (43°07’14.63’’ S, 73°44’25.72’’ W), a coastal bay influenced by open sea currents from Guafo’s mouth. The data (0 to -10 meters deep) cover the period from June 2017 to May 2018, overlapping with sampling dates of the reciprocal transplant study described by Yévenes et al. (2025) (49). The data were processed, projected, and visualized using Ocean Data View ODV v5.32 software (https://odv.awi.de/). Descriptive statistics (mean ± standard deviation) were calculated for each environmental variable and site to characterize temporal variability and are provided in the Supplementary Material **Supplementary Table 1**.

### 2.2 Data and lncRNA database

This study utilized clean reads from 12 cDNA libraries sequenced by RNA-Seq, which are available in the Sequence Read Archive (SRA) in GenBank (BioProject accession number PRJNA630273). These libraries originated from mussels that underwent a reciprocal transplant experiment previously published (49). These libraries correspond to the same biological samples previously analyzed, which are here reanalyzed to investigate lncRNA expression patterns. They include three biological replicates of the self-transplanted (A) and cross-transplanted (T) groups of mussels, collected in Cochamó and Yaldad on July 28th, 2018, when the experiment ended after 91 days.

Briefly, each biological replicate was constructed by combining equal molar amounts of total RNA from five individual gill tissue extractions. RNA integrity and purity were assessed using agarose gel electrophoresis and an Agilent TapeStation 2200 system, and only samples with RNA integrity number (RIN) values above 9 were retained for library preparatiion. Each biological replicate corresponds to a pooled sample of five individuals, and the effective sample size for statistical inference is the number of pooled replicates (n = 3 per condition). The cDNA libraries, prepared with the TrueSeq Stranded mRNA LT Sample Prep Kit, were sequenced on the Illumina HiSeq 4000 platform with a 100 bp paired-end approach.

The 43,011 non-coding sequences from the *M. chilensis* lncRNA database (Mch_lncRNAdb), as documented in Yévenes et al. (2024) (34), were used as a reference for mapping. Contigs with annotations, transcripts with open reading frames (ORFs) and sequences with coding potential were excluded from this database. While Mch_lncRNAdb provides a reference for *M. chilensis* lncRNAs, it may not capture the full diversity of lncRNA transcripts in this species; therefore, the present results should be interpreted within the scope of this reference dataset.

### 2.3 Differential expression analysis

The CLC Genomic Workbench (CLCgw) v25.0.1 (Qiagen Bioinformatics™) platform was used for mapping, normalizing, and quantifying the clean reads with tools from the RNA-Seq analysis suite. Gene expression levels were estimated as transcript per million (TPM), a normalization approach that accounts for differences in sequencing depth (library size) and gene length across samples, thereby enabling robust comparison of transcript abundance. The lncRNA expression levels were determined by globally aligning the reads to the Mch_lncRNAdb sequences. Various filters were applied during read mapping to ensure robustness and minimize biases in the alignments. These filters included a mismatch cost of 2, a maximum insertion and deletion cost of 3, length and similarity fractions set at 0.8, and a maximum limit of 10 hits per read. Transcripts with invalid values or zero read counts were excluded. Differential expression was analyzed using a negative binomial generalized linear model (GLM), with the Wald test applied to assess whether differences significantly deviated from zero. This modeling framework accounts for variability in count-based RNA-seq data while incorporating normalization for library size and biological replication. Fold change (FC) values were estimated within the GLM framework, ensuring consistency between effect size estimation and statistical testing. Future analyses using alternative frameworks with explicit dispersion moderation and shrinkage (e.g., DESeq2 or edgeR) could further assess the robustness of the results. Two different filtering approaches were employed to explore differential long non-coding RNA (lncRNA) expression. To prioritize robust and biologically meaningful signals, statistical significance (FDR < 0.05) was combined with fold change thresholds (|FC| ≥ 3). Considering the large number of transcripts evaluated, this combined criterion was used to balance the detection of consistent expression differences while reducing the likelihood of false positives. More restrictive thresholds (e.g., Bonferroni correction or higher fold change cutoffs) were explored but resulted in the loss of detectable differentially expressed lncRNAs.

The RNA-Seq results from the 12 sequenced cDNA libraries were visualized with a clustered heatmap, organized by replicate, sample (A and T), and location, using Euclidean distances and average linkage for clustering. Additionally, differential gene expression across replicates was statistically evaluated with principal component analysis, and the relationship between -log_10_(FDR p-value) and log_2_(FC value) by location was displayed using a volcano plot.

Venn diagrams were used to facilitate sample comparisons, enabling the identification and selection of lncRNAs that met the filtering criteria. Comparisons of FC values between samples were based on the δ_LF_ and δ_HA_ criteria, using the comparison of self-transplanted from Cochamó versus self-transplanted from Yaldad (ACo vs. AYa) as the reference. According to the δ_LF_ criterion, FC values were evaluated between cross-transplanted individuals from Yaldad and Cochamó auto-transplanted individuals (TYa vs. ACo), as well as between cross-transplanted individuals from Cochamó and Yaldad auto-transplanted individuals (TCo vs. AYa). Additionally, under the δ_HA_ criterion, FC values were compared between cross-transplanted individuals from Cochamó and their Cochamó auto-transplanted counterparts (TCo vs. ACo), and between cross-transplanted individuals from Yaldad and their Yaldad auto-transplanted counterparts (TYa vs. AYa). These criteria were used as a conceptual framework to identify expression patterns consistent with local adaptation. Although they do not directly measure fitness or adaptive performance, they represent a widely accepted approach for evaluating genotype-environment responses in reciprocal transplant studies.

Subsequently, a more stringent threshold for FC value was applied to reduce the likelihood of false positives from multiple comparisons. These stricter thresholds included an FC value ≥ |10| and an FDR p-value < 0.05, aiming to identify lncRNAs with high and significant fold changes. The goal of applying this filter was to highlight lncRNAs with potentially striking differences in the biological context being explored, even though it carries the risk of excluding lncRNAs with lower fold changes that may still have crucial biological relevance. Identifying these last kinds of lncRNAs can be challenging, as their expression levels might closely resemble those of genes with less significant biological impact.

To address this challenge, alternative analytical methods, such as cloning, could be used to ensure that biologically relevant lncRNAs are not overlooked. This stringent threshold was used to prioritize lncRNAs with the most pronounced transcriptional differences across comparisons, thereby reducing the likelihood of emphasizing weak signals and facilitating the identification of robust candidate transcripts for downstream analyses.

Since the number of differentially expressed lncRNAs (DE-lncRNAs) detected under both δ_LF_ and δ_HA_ criteria may reflect patterns consistent with differential genotype-environment responses, these metrics were counted and plotted to examine whether gene expression changes were widespread and balanced or concentrated in a subset of candidate lncRNAs. These DE-lncRNAs were identified as significant, and their sequences were extracted, annotated, and functionally categorized.

### 2.4 Differentially expressed lncRNAs annotations

The annotation and functional categorization of sequences identified as DE-lncRNAs in *M. chilensis* were conducted using available lncRNA databases, based on sequence similarity, database annotation, and predicted features, thereby providing an inferential framework to identify potential biological associations and relevance. While not directly demonstrating lncRNA function, this approach enables the identification of candidate regulatory elements for further investigation and offers a robust basis for generating biologically informed hypotheses in non-model systems.

For example, iLoc-LncRNA (66) was used to predict the subcellular localization of DE-lncRNAs (http://lin-group.cn/server/iLoc-LncRNA/predictor.php), while RNAcentral (https://rnacentral.org/) was employed to identify similar sequences. The latter provided information on matching hits, including e-values and percent identity with homologous lncRNAs from the closely related species *M. galloprovincialis*. Outputs from RNAcentral also enabled the identification (names and aliases) of these DE-lncRNAs and provided links to additional databases such as LncBook (https://ngdc.cncb.ac.cn/lncbook/home/), NONCODE (http://www.noncode.org/index.php/), e!Ensembl (https://www.ensembl.org/index.html/), GeneCards (https://www.genecards.org/), and LNCipedia (https://lncipedia.org/), which offer information on lncRNA classes and associated ontological terms described in other species. These annotations were used as inferential descriptors for DE-lncRNAs based on sequence similarity, ontology class, and predicted subcellular localization.

Overall, the annotation strategy employed here relies on publicly available databases and predictive tools, which are largely derived from model and non-marine organisms. When available, annotations were reported using the most specific protein names supported by sequence similarity, together with their corresponding database accession numbers, whereas in cases where precise functional assignment was not possible, descriptions are provided at the domain or protein family level, reflecting the limitations inherent to non-model species. As a result, these resources are inherently inferential and may be influenced by taxonomic biases, yet they provide a valuable framework for contextualizing DE-lncRNAs and generating biologically informed hypotheses in non-model systems such as *M. chilensis*.

### 2.5 Genome mapping of DE-lncRNAs and neighboring protein coding genes

The genomic positions of the identified differentially expressed long non-coding RNAs (DE-lncRNAs) were determined by mapping them against the whole-genome sequence of *M. chilensis* (46) using CLCgw software. The mapping results were exported in SAM format and uploaded to the GALAXY online server (https://usegalaxy.org/) (67). These files were then converted into interval, BED, and GFF formats for further annotation of lncRNAs in the genome. The annotation extraction tool in CLCgw identified NPC-genes flanking up to 10 kb upstream and downstream of the DE lncRNA in the samples.

### 2.6 Expression comparison between DE-lncRNAs-linked NPC-genes and candidate adaptive differentially expressed genes

The already published information was considered to gain insights into the expression of NPC-genes linked to DE-lncRNAs detected in this study’s comparisons. Yévenes et al. (2025) (49) identified 3687 differentially expressed adaptive candidate genes (caDEGs) from whole transcriptomes (FC value ≥ |4| and FDR p-value≤ 0.05), found under both δ_LF_ and δ_HA_ criteria for testing local adaptations in the same individuals from Cochamó and Yaldad used in this study. Venn diagrams were used to facilitate this comparison, helping to identify NPC-genes of DE-lncRNAs that match with caDEGs.

## 3 Results

### 3.1 Environmental characterization

The marine environment within the inner sea of Chiloé Island exhibits differences between its northern and southern zones. These differences vary seasonally and are influenced by climatic conditions, marine currents, and seawater properties. In the north, average salinity levels of 21.2 ± 1.86 ppt and sea surface temperatures of 17 ± 0.72 °C have been recorded during summer, with temperatures dropping to 11.3 ± 0.45 °C in winter. In contrast, the south has higher salinity levels of 31.6 ± 0.46 ppt and summer temperatures of 21.7 ± 5.16 °C, which decrease to 11.5 ± 1.06 °C in winter (68).

The reciprocal transplant experiment lasted 91 days, starting on April 26th, 2018. At the beginning, Cochamó had a seawater surface temperature of 12.2 °C, salinity of 19.0 ppt, and pH of 6.8. In contrast, the seawater surface at Yaldad showed a temperature of 11.6 °C, salinity of 31.4 ppt, and pH of 7.05. By the end of the experiment on July 28th, 2018, changes in oceanographic parameters were observed. In Cochamó, the sea surface temperature dropped to 10.4 °C, salinity increased to 20.5 ppt, and the pH slightly rose to 7.02. Meanwhile, Yaldad showed a sea surface temperature of 9.6 °C, salinity of 32.0 ppt, and a pH of 7.62.

**Figure 1:**
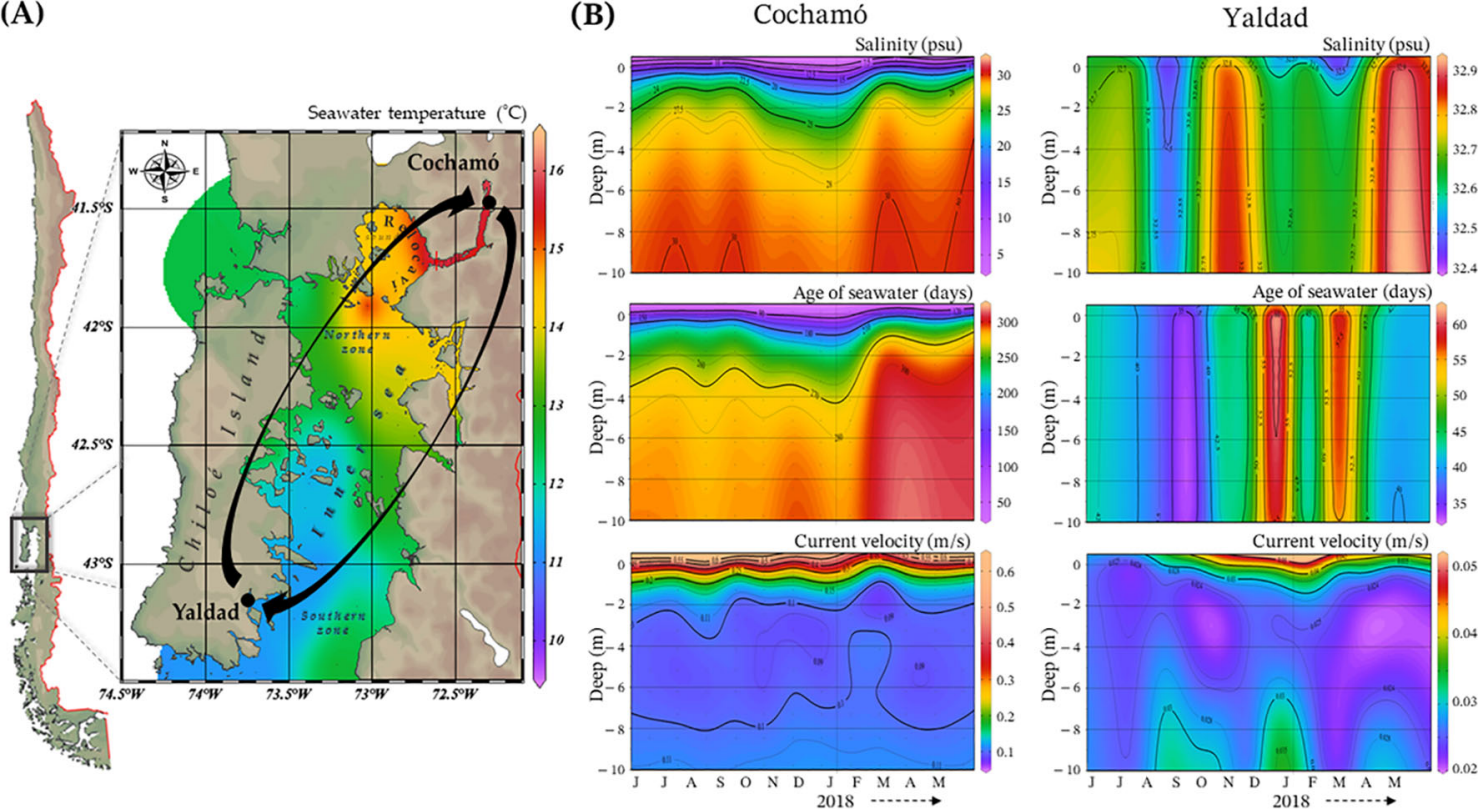
Sampling locations and environmental conditions map. Map **(A)** with the geographic location of the sampled natural seedbeds of *Mytilus chilensis*, Cochamó (north), and Yaldad (south) of Chiloé Island. Mean seawater temperature (colored background) between June 2017 and May 2018. The red arrows represent the reciprocal transplant experiment. **(B)** Mean seawater of salinity, age of seawater, and currents for each sampling location. The scaling of the parameters is different between locations.

The oceanographic data from the CHONOS database for Cochamó and Yaldad, collected at depths from 0 to -10 meters, covered the period from June 2017 to May 2018, coinciding with the sampling dates of the reciprocal transplant experiment (April – June 2018; **Figure 1**). During this period, on average, Cochamó exhibited lower salinity (25.45 ± 6.32 psu) and higher current velocity (0.155 ± 0.11 m/s) compared to Yaldad (32.72 ± 0.12 psu and 0.027 ± 0.005 m/s, respectively), while temperature was similar between sites, 11.61 ± 1.23 °C for Cochamó and 11.16 ± 1.45 °C for Yaldad (values expressed as mean ± standard deviation). Additionally, water age was substantially higher in Cochamó (252.7 ± 65.1 days) than in Yaldad (44.4 ± 7.7 days). These data show that Cochamó and Yaldad differ in environmental conditions within the inner sea of Chiloé Island, with Cochamó showing higher marine currents, and longer water retention times than Yaldad, but lower salinity. These differences in environmental variability provide important context for interpreting the contrasting transcriptomic responses observed between sites.

In addition to these differences in central tendencies, as reflected by the average patterns shown in **Figure 1**, CHONOS data showed temporal variability across all measured variables. This variability was summarized using descriptive statistics (mean ± SD) for each site and variable, as presented in **Supplementary Table 1**, providing additional context for the environmental conditions experienced during the experimental period.

### 3.2 Mapping of clean reads

**Table 1** shows the number of clean reads from individuals in Cochamó and Yaldad under self- and cross-transplantation, using Mch_lncRNAdb sequences as a reference for mapping. Among self-transplanted Cochamó individuals (ACo), 5.02% of the 54.3 million reads (standard deviation SD = 4.1 million) mapped to the Mch_lncRNAdb, compared with 5.18% of the 33.1 million reads (SD = 1.1 million) in cross-transplanted individuals (TCo). Similarly, 5.03% of 55.6 million reads (SD = 677,061) from self-transplanted Yaldad individuals (AYa) mapped to Mch_lncRNAdb, while the percentage was 5.06% for cross-transplanted individuals (TYa), with 32.9 million reads (SD = 1.1 million).

**Table 1:** Mapping read characteristics.

A)	Parameter	Cochamó					
		Auto-transplanted			Cross-transplanted		
		Media	SD	%	Media	SD	%
	Total of clean reads	54260670.67	4114336.26	100.00	33079882.00	1061904.06	100.00
	Mapped reads	2723343.33	214801.69	5.02	1713142.00	50120.05	5.18
	Reads not mapped	51537327.33	4018983.08	94.98	31366740.00	1013552.31	94.82
B)	Parameter	Yaldad					
		Auto-transplanted			Cross-transplanted		
		Media	SD	%	Media	SD	%
	Total of clean reads	55553940.67	677060.52	100.00	32849423.00	2932647.81	100.00
	Mapped reads	2791765.33	76464.45	5.03	1663086.33	118392.77	5.06
	Reads not mapped	52762175.33	603528.50	94.97	31186336.67	2814274.81	94.94

Summary of mean values, standard deviations (SD), and percentage (%) of mapped reads from Illumina RNA-Seq data for self- and cross-transplanted *Mytilus chilensis* individuals from two natural seedbeds in the inner sea of Chiloé Island: Cochamó (A) and Yaldad (B).

### 3.3 Differential expression analysis of lncRNAs

**Figure 2:**
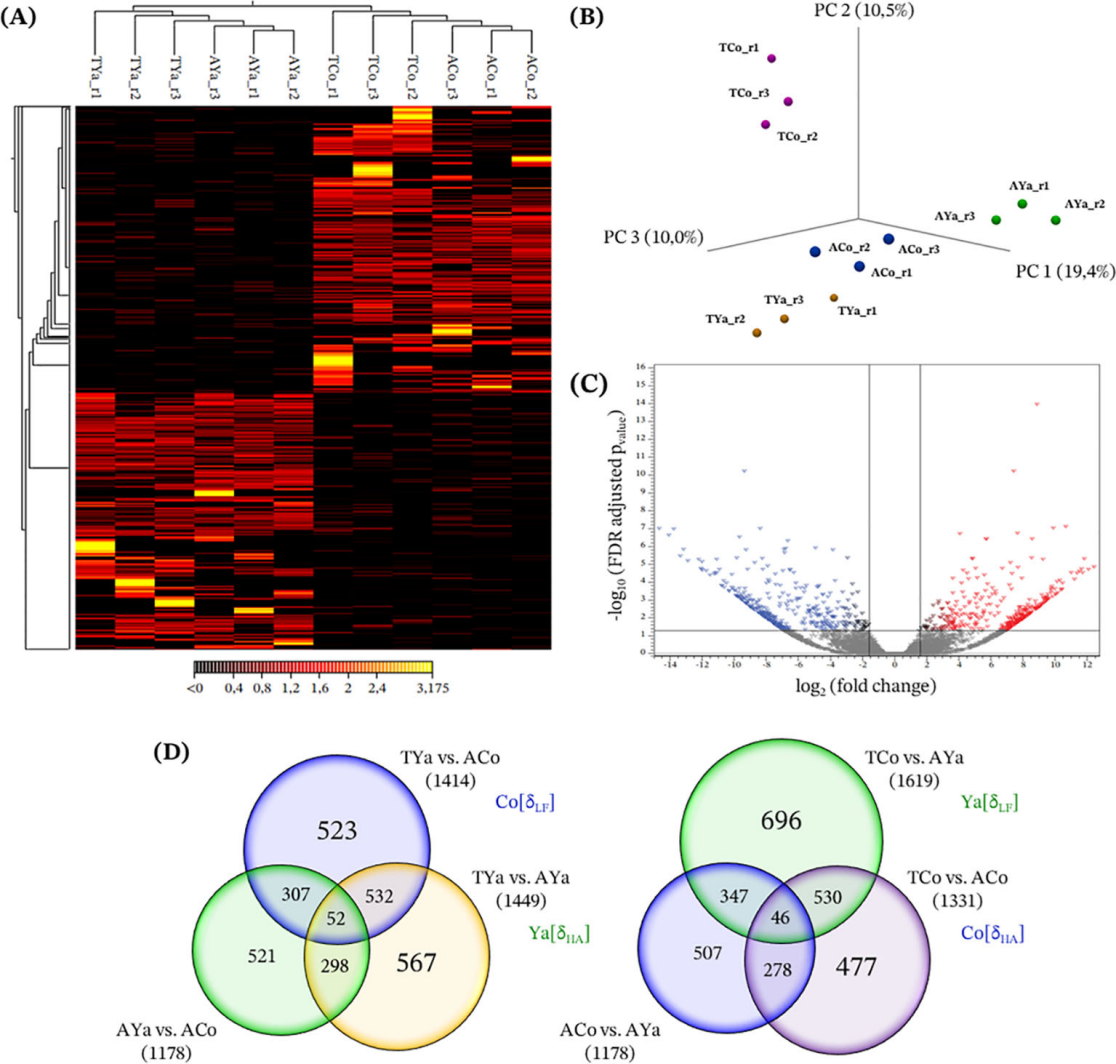
Differential expression analysis of lncRNAs. **(A)** Heatmap with hierarchical clustering of differentially expressed lncRNAs (DE-lncRNAs), primarily structured by location with additional within-group variation associated to transplantation. Each row represents a lncRNA and each column corresponds to differential gene expression by replicate (r1, r2 and r3) in self- and cross- transplanted individuals from Cochamó (ACo, TCo) and Yaldad (AYa, TYa). The color scale indicates normalized expression levels, ranging from low (black) to high (yellow/red). **(B)** Principal component analysis (PCA) based on lncRNA expression profiles of replicated samples from Cochamó and Yaldad, showing separation of biological replicates according to experimental condition. ACo and AYa represent self-transplanted individuals from Cochamó and Yaldad, respectively, while TCo and TYa correspond to cross-transplanted individuals between these locations. **(C)** A volcano plot pointing out lncRNAs with significantly different expression levels (DE-lncRNAs) between Cochamó and Yaldad samples, with significance thresholds (FDR adjusted p-value < 0.05 and log_2_(fold change) ≥ 3) indicated by horizontal and vertical reference lines. **(D)** Venn diagrams illustrating the overlap of DE-lncRNAs among pairwise comparisons of experimental self- and cross- transplanted conditions. Numbers indicate exclusive and shared lncRNAs across the comparisons using a lenient filter of FCvalue ≥|3| and FDR p-value ≤0.05.

The lenient filters (FC value ≥ |3| and FDR p-value ≤ 0.05) revealed significant differences in lncRNAs expression profiles between samples (**Figure 2**). Euclidean distances between expression values showed that the lncRNAs clustered into two main groups in the heatmap (**Figure 2A**), with some highly expressed in Cochamó individuals (ACo and TCo) but not in Yaldad (AYa and TYa), showing differences between locations. These patterns indicate that the primary clustering structure is driven by location (Cochamó vs. Yaldad), with ACo and TCo grouping together and AYa and TYa forming a separate cluster. However, within each location, self- and cross-transplanted samples also show consistent sub-structuring, suggesting that transplant condition contributes to variation in expression profiles. While the heatmap distinguishes samples by location, differences between self- and cross-transplanted individuals are also observable within each group, although these are less pronounced than the location effect. The principal component analysis (PCA) further illustrated the variability in lncRNA expression, separating samples by replicates and groups. In the three-dimensional scatterplot (**Figure 2B**), principal components 1, 2, and 3 explained 19.4%, 10.5%, and 10% of the variability, respectively, forming four distinct groups consistent with the heat map results. The volcano plot (**Figure 2C**) was consistent with these patterns, showing up-regulated (red) lncRNAs. Most expressed lncRNAs clustered near the origin, while several showed extreme log_2_(FC values) and high statistical significance [-log_10_(FDR p-values)], consistent with differences observed between transplant conditions.

Venn diagrams illustrate the number of DE-lncRNAs in self- and cross-transplanted Cochamó and Yaldad individuals, highlighting those exclusive to each group with significant fold changes (**Figure 2D**). Using the δ_LF_ criterion for Cochamó, 1414 DE-lncRNAs were identified between TYa and ACo individuals, with 523 exclusives to this comparison. In Yaldad (TCo vs. AYa), 1619 DE-lncRNAs were found, with 696 being exclusive. Under the δ_HA_ criterion for Cochamó (TCo vs. ACo) and Yaldad (TYa vs. AYa), 1331 and 1419 DE-lncRNAs were identified, with 477 and 567 being exclusive, respectively. Collectively, these Venn diagrams, heatmap, PCA, and volcano plots provide an overview of the significant DE-lncRNAs that distinguish Cochamó and Yaldad individuals.

### 3.4 Patterns of DE-lncRNAs under δ_LF_ and δ_HA_ criteria

**Figure 3:**
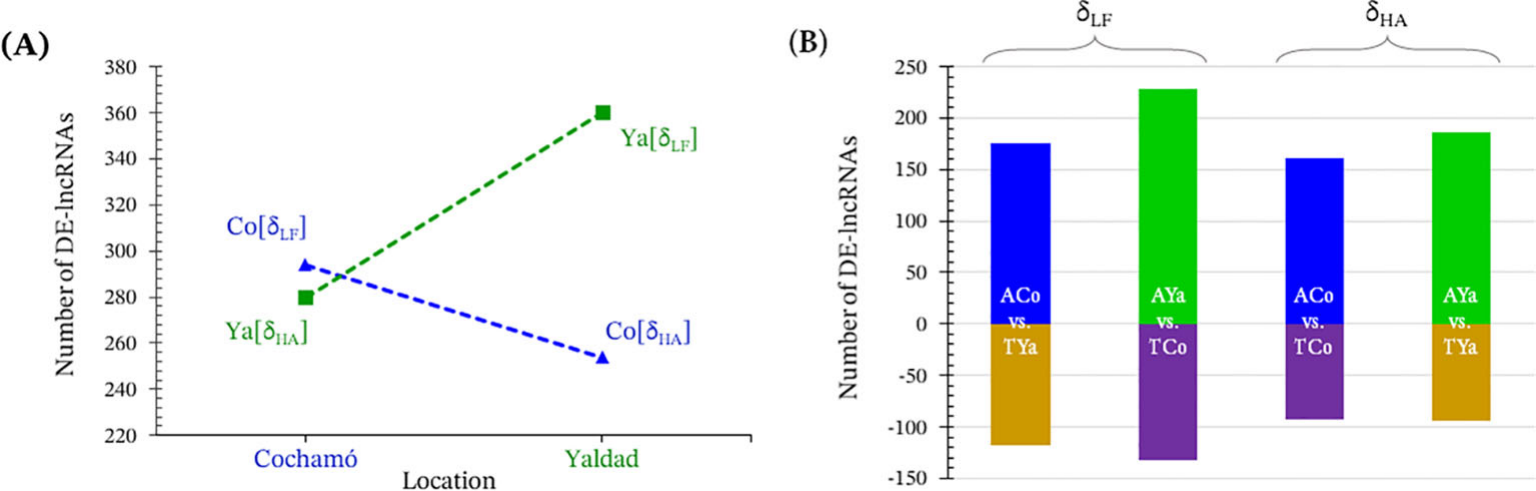
Local adaptation patterns and differential expressed lncRNAs (DE-lncRNAs). **(A)** Comparison of the number of differentially expressed lncRNAs (DE-lncRNAs) in Cochamó (Co) and Yaldad (Ya) using a FC ≥|10| and FDR p-value ≤0.05, under the local vs. foreign (δ_LF_) and home vs. away (δ_HA_) criteria for testing local adaptation. Blue triangles represent Cochamó and green squares represent Yaldad. **(B)** Bar plots showing the number of exclusive DE-lncRNAs for each sample, with transplanted samples as negative values. Colors indicate self-transplanted individuals from Cochamó (ACo, blue) and Yaldad (AYa, green), and transplanted individuals from Cochamó (TCo, purple) and Yaldad ((TYa, brown).

The Venn diagram shows the number of DE-lncRNAs in Cochamó and Yaldad samples, as well as those unique to the δ_LF_ (523 for TYa vs. ACo, and 696 for TCo vs. AYa) and δ_HA_ criteria (477 for TCo vs. ACo and 567 for TYa vs. AYa) in samples from Cochamó and Yaldad. A comparative analysis was conducted using strict filters (FC value ≥ |10|, FDR p-value ≤ 0.05). This analysis revealed a distinct pattern of differential expression, with Yaldad individuals exhibiting greater differences than those from Cochamó under both criteria (**Figure 3A**). Overall, Cochamó showed 294 exclusive DE-lncRNAs for the δ_LF_ criterion (TYa vs. ACo); 176 were upregulated in ACo and 118 in TYa (**Figure 3B**). Yaldad (TCo vs. AYa) showed 360 exclusive DE-lncRNAs, with 228 up-regulated in AYa and 132 in TCo. For the δ_HA_ criterion, Cochamó (TCo vs. ACo) showed 254 exclusive DE-lncRNAs, with 161 up-regulated in ACo and 93 in TCo. In comparison, Yaldad (TYa vs. AYa) showed 280 exclusive DE-lncRNAs, with 186 up-regulated in AYa and 94 in TYa.

### 3.5 Genome mapping of DE-lncRNAs

**Figure 4:**
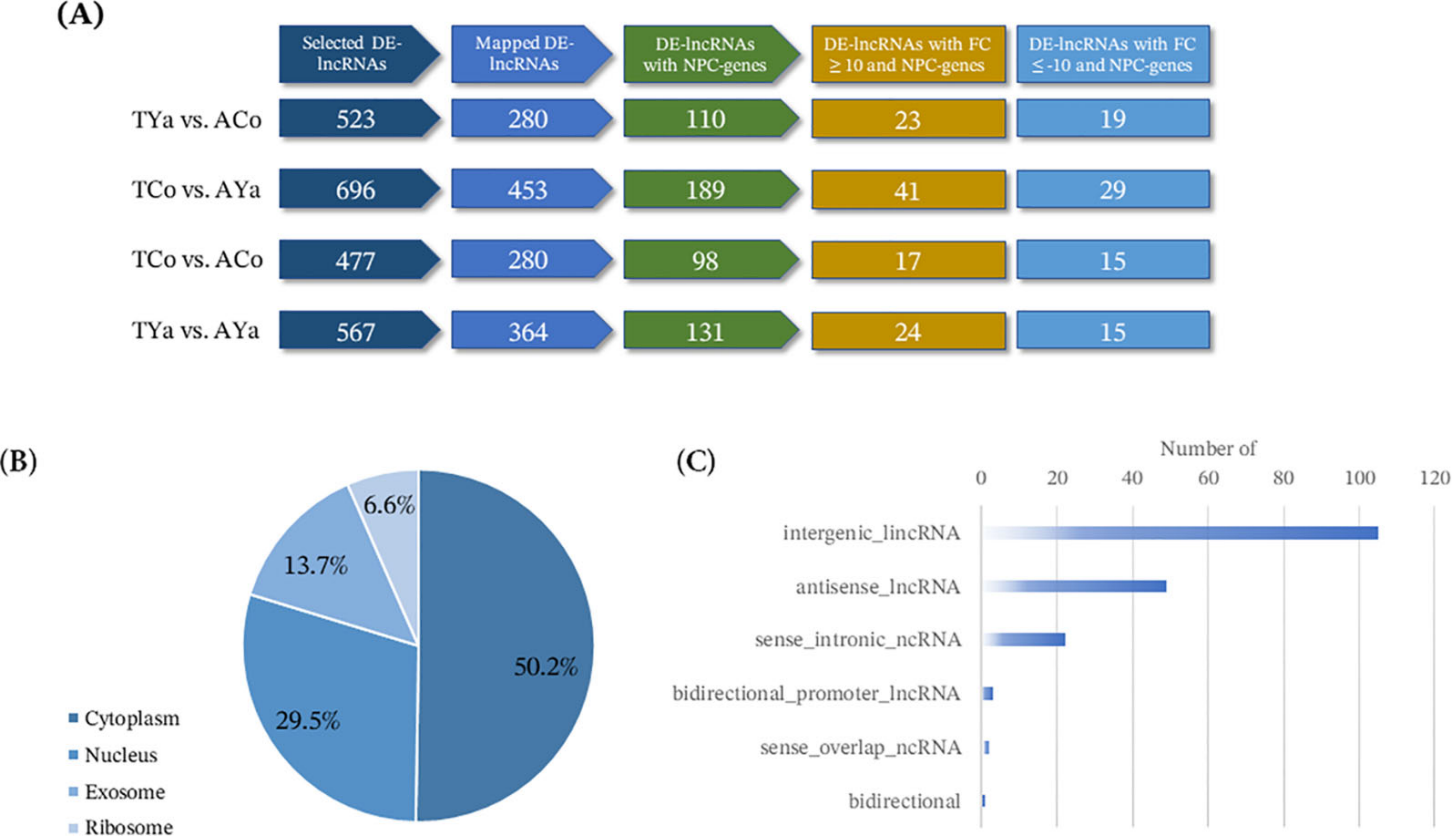
Differentially expressed lncRNAs (DE-lncRNAs) annotations. **(A)** Flowchart summarizing the progressive filtering of differentially expressed lncRNAs (DE-lncRNAs) under δ_LF_ and δ_HA_ criteria. The diagram illustrates the sequential reduction from the initial set of identified mapped DE-lncRNAs, DE-lncRNAs associated with NPC genes, and the final subset with extreme expression changes (|fold change| ≥ 10). Numbers indicate the count of lncRNAs retained at each step. **(B)** Pie chart showing the predicted distribution of DE-lncRNAs across cellular compartments and **(C)** Bar chart showing the proportion of DE-lncRNAs according to their ontogenetic categories.

Mapping the DE-lncRNA sequences detected in self- and cross-transplanted individuals from Cochamó and Yaldad against the chromosome-level reference genome of *M. chilensis* revealed widespread chromosomal distribution, enabling the identification of their NPC-genes located within 10 Kb upstream and downstream. In this context, a progressive decrease in the number was observed as more stringent filters were applied (**Figure 4A**). For example, among the 523 DE-lncRNAs detected in the TYa vs. ACo comparison for Cochamó under the δ_LF_ criterion, 280 were mapped and linked to 140 NPC-genes.

When restricting the analysis to DE-lncRNAs that have NPC-genes, the count drops to 110, and only a small number showed differential expression with high fold change values, either with a fold change ≥ 10 (23 for ACo) or fold change ≤ −10 (19 for TYa). Likewise, out of the 696 DE-lncRNAs identified for Yaldad in the TCo vs. AYa comparison, 453 were mapped, and 255 NPC-genes were identified; this number decreased to 189 when focusing only on lncRNAs with NPC-genes. Higher fold change values were observed in 41 DE-lncRNAs for AYa samples with fold change ≥ 10 and 29 for TCo samples with fold change ≤ −10. A similar trend was seen under the δ_HA_ criterion. In the TCo vs. ACo comparison, 477 DE-lncRNAs were found, of which 280 were mapped and 131 NPC-genes. Additionally, 98 DE-lncRNAs with NPC-genes were identified, with 17 (ACo) and 15 (TCo) showing fold changes of ≥ 10 and ≤ −10, respectively. Finally, in the TYa vs. AYa comparison, 567 DE-lncRNAs were identified, with 364 mapped and 189 NPC-genes. When focusing only on DE-lncRNAs with NPC-genes, the count dropped to 131, including 24 (AYa) and 15 (TYa) with fold changes of ≥ 10 and ≤ −10, respectively. Overall, these patterns indicate that although the initial number of DE-lncRNAs was high, only a small portion could be mapped and linked to NPC-genes. Within this subset, a smaller proportion showed the highest transcriptional changes, representing a subset of candidate DE-lncRNAs for further analysis.

### 3.6 Annotation of DE-lncRNA

Different web databases provided information on homologous sequences based on database annotation of the 183 DE-lncRNAs with linked NPC-genes that showed the highest expression changes (fold change ≥ 10 and ≤ −10). According to their predicted subcellular localization (**Figure 4B**), most of these DE-lncRNAs were predicted to be located in the cytoplasm and cytosol (92), followed by the nucleolus, nucleus, and nucleoplasm (54). At the same time, smaller proportions were annotated to exosomal (25) and ribosomal (12) compartments.

This distribution shows that DE-lncRNAs with NPC-genes are predicted to be associated with both nuclear and cytoplasmic compartments. Similarly, the ontological classification (**Figure 4C**) of these DE-lncRNAs with NPC-genes showed that most belong to long intervening/intergenic ncRNAs (lincRNAs) (57.4%), followed by antisense lncRNAs (26.8%) and, to a lesser degree, sense intronic ncRNAs (12%). Minor categories included bidirectional promoter lncRNAs (1.6%), sense overlap ncRNAs (1.1%), and a single bidirectional transcript (0.5%). This distribution indicates that most DE-lncRNAs with NPC-genes correspond to intergenic intervening ncRNAs (lincRNAs), while antisense and intronic transcripts represent smaller proportions.

#### 3.6.1 Local vs. foreign (δ_LF_) criterion comparison of DE-lncRNAs

**Table 2** summarizes information for the δ_LF_ criterion for Cochamó and Yaldad regarding the top ten DE-lncRNAs with the highest FC value linked to NPC-genes. The **Table 2** includes predicted subcellular localization and the ontology class of each DE-lncRNA. It also shows the number of hits from the search for lncRNAs in the RNACentral database and the number of homologous sequences found in the sister species *Mytilus galloprovincialis* (Mga). Approximately 70% of the identified *M. chilensis* DE-lncRNAs showed sequence similarity to Mga, with an average e-value of 1.2E + 04 and an average identity of 63.6%. In most cases, the percentage of identity with Mga homologous sequences did not exceed 71%. However, one exception is the 83.6% identity (e-value=5.9E-61) of the intergenic DE-lincRNA Contig_0156322 (FC value=-67) in TCo samples from the TCo vs AYa comparison, which was predicted to be localized in the nucleus.

**Table 2:** Local vs. foreign (δ_LF_) criterion comparison of DE-lncRNAs.

	Comparison	Samples	lncRNA Contig ID	Fold change	Subcellular localization	RNAcentral hits	LNCPedia sequence ontology class	RNACentral Mga Hits	RNACentral Mga ID	E-value	Identity (%)
(A)	TYa vs. ACo	ACo	Contig_0079722_	744.70	Exosome	770	intergenic_lincRNA	–	–	–	–
			Contig_0005731_	338.22	Exosome	767	intergenic_lincRNA	3	URS00021A2660_29158	9.1.E+03	63
			Contig_0043086_	126.59	Cytoplasm	757	sense_intronic_ncRNA	4	URS000219B639_29158	1.2.E+03	53
			Contig_0103978_	88.89	Cytoplasm	757	intergenic_lincRNA	6	URS00021B4C10_29158	1.8.E+03	67
			Contig_0112889_	86.45	Nucleolus, Nucleus	684	intergenic_lincRNA	6	URS000218B635_29158	1.1.E+05	71
			Contig_0032387_	61.43	Cytoplasm	794	antisense_lncRNA	3	URS00021BF3E6_29158	3.3.E+03	65
			Contig_0103376_	33.90	Cytoplasm	817	antisense_lncRNA	–	–	–	–
			Contig_0114921_	32.48	Nucleolus, Nucleus	453	intergenic_lincRNA	1	URS00021AEE28_29158	2.7.E+04	67
			Contig_0078203_	28.27	Cytoplasm	807	sense_intronic_ncRNA	6	URS00021B6008_29158	1.0.E+04	59
			Contig_0055100_	25.72	Exosome	783	sense_intronic_ncRNA	7	URS000219793E_29158	1.0.E+04	55
		TYa	Contig_0035749_	-139.32	Exosome	731	intergenic_lincRNA	33	URS000218B4C4_29158	2.7.E+03	59
			Contig_0145142	-64.19	Cytoplasm	229	antisense_lncRNA	–	–	–	–
			Contig_0100418_	-48.18	Cytoplasm	810	intergenic_lincRNA	4	URS00021D3B65_29158	1.5.E+04	61
			Contig_0161780	-33.86	Nucleolus, Nucleus	379	intergenic_lincRNA	–	–	–	–
			Contig_0172359	-32.41	Nucleolus, Nucleus	80	intergenic_lincRNA	1	URS00021A3AED_29158	2.6.E+04	66
			Contig_0049013_	-31.92	Cytoplasm	800	sense_intronic_ncRNA	43	URS00021B11C6_29158	1.9.E+01	65
			Contig_0169973	-30.75	Nucleolus, Nucleus	208	intergenic_lincRNA	–	–	–	–
			Contig_0128661	-29.46	Cytoplasm	504	intergenic_lincRNA	–	–	–	–
			Contig_0035302_	-26.07	Cytoplasm	776	sense_intronic_ncRNA	2	URS00021CCC02_29158	1.5.E+03	59
			Contig_0036314_	-19.17	Cytoplasm	777	intergenic_lincRNA	8	URS00021AB2DB_29158	5.2.E+03	58
(B)	TCo vs. AYa	AYa	Contig_0139356	541.71	Cytoplasm	662	bidirectional_lncRNA	–	–	–	–
			Contig_0122642_	472.28	Cytoplasm	551	sense_intronic_ncRNA	–	–	–	–
			Contig_0017531_	270.84	Exosome	725	intergenic_lincRNA	–	–	–	–
			Contig_0091214_	268.52	Exosome	708	intergenic_lincRNA	2	URS000218C478_29158	1.80E+04	56
			Contig_0112788_	250.40	Nucleolus, Nucleus	789	sense_intronic_ncRNA	218	URS00021B397B_29158	8.60E+03	68
			Contig_0130092	210.82	Cytoplasm	746	sense_intronic_ncRNA	4	URS00021AEEE0_29158	2.30E+04	65
			Contig_0145936	199.75	Nucleolus, Nucleus	196	sense_intronic_ncRNA	–	–	–	–
			Contig_0012027_	187.99	Cytoplasm	743	intergenic_lincRNA	7	URS00021C0E1D_29158	8.5.E+03	71
			Contig_0107622_	105.91	Nucleolus, Nucleus	564	intergenic_lincRNA	4	URS00021DF27E_29158	1.3.E+04	68
			Contig_0005745_	102.25	Nucleolus, Nucleus	751	sense_intronic_ncRNA	–	–	–	–
		TCo	Contig_0003123_	-400.71	Nucleolus, Nucleus	757	intergenic_lincRNA	9	URS00021C5ACA_29158	5.4.E+02	62
			Contig_0156322	-67.12	Nucleolus, Nucleus	281	intergenic_lincRNA	3	URS000218F37F_29158	5.9.E-61	83
			Contig_0085257_	-57.51	Cytoplasm	608	intergenic_lincRNA	4	URS00021DE537_29158	2.3.E+04	58
			Contig_0002892_	-57.47	Cytoplasm	784	intergenic_lincRNA	12	URS00021ECE47_29158	2.6.E+00	65
			Contig_0158906	-55.86	Cytoplasm	149	intergenic_lincRNA	–	–	–	–
			Contig_0008086_	-43.45	Cytoplasm	815	sense_intronic_ncRNA	3	URS00021CAB63_29158	6.4.E+03	63
			Contig_0087620_	-42.14	Nucleolus, Nucleus	660	sense_intronic_ncRNA	54	URS00021E1715_29158	2.1.E+02	62
			Contig_0046920_	-35.63	Exosome	782	intergenic_lincRNA	2	URS00021E09C3_29158	5200	65
			Contig_0158461	-32.12	Nucleolus, Nucleus	310	antisense_lncRNA	2	URS00021DA27E_29158	3.4.E+03	68
			Contig_0031091_	-30.92	Cytoplasm	824	antisense_lncRNA	4	URS00021A2F21_29158	2600	60

Results of the annotations for top ten up-regulated lncRNAs (DE-lncRNAs) detected in each sample comparison under the local vs. foreign (δ_LF_) criterion for Cochamó (A) and Yaldad (B). Labels: ACo and TCo, self- and cross- transplanted individuals from Cochamó; respectively. Likewise, AYa and TYa, self- and cross- transplanted individuals from Yaldad; Mga, *Mytilus galloprovincialis*; Fold change values of transplanted individuals are represented as negative numbers.

#### 3.6.2 Home vs. away (δ_HA_) criterion comparison of DE-lncRNAs

Similarly, **Table 3** lists the top ten DE-lncRNAs selected by FC value according to the δ_HA_ criterion for Cochamó and Yaldad. The table shows that 83% of the DE-lncRNA sequences show sequence similarity to Mga, with an average e-value of 1.6E + 04 and an average identity of 64.6%. Among these, the sense intronic DE-ncRNA Contig_0071475 (FC value = -41) was identified in TCo samples from the TCo vs. ACo comparison, which showed 97.1% identity (e-value = 1.5E-73) and was predicted to be localized in the cytoplasm.

**Table 3:** Home vs. away (δ_HA_) Criterion Comparison of DE-lncRNAs.

	Comparison	Samples	lncRNA Contig ID	Fold change	Subcellular localization	RNAcentral hits	LNCpedia sequence ontology class	RNAcentral Mga hits	RNAcentral Mga ID	E-value	Identity (%)
(A)	TCo vs. ACo	ACo	Contig_0100429_	109	Cytoplasm	795	intergenic_lincRNA	3	URS00021BE419_29158	8.0.E+04	67
			Contig_0115849_	102	Nucleolus, Nucleus	558	intergenic_lincRNA	–	–	–	–
			Contig_0104183_	59	Cytoplasm	780	antisense_lncRNA	2	URS0002190CD9_29158	1.0.E+03	70
			Contig_0168455	53	Nucleolus, Nucleus	112	intergenic_lincRNA	1	URS000218A940_29158	1.0.E+05	61
			Contig_0055100_	31	Exosome	783	sense_intronic_ncRNA	7	URS000219793E_29158	1.0.E+04	55
			Contig_0069732_	31	Nucleolus, Nucleus	683	intergenic_lincRNA	2	URS00021E9701_29158	1.9.E+03	65
			Contig_0026577_	22	Nucleolus, Nucleus	825	sense_intronic_ncRNA	9	URS00021E4DDF_29158	3.9.E-03	61
			Contig_0044964_	21	Exosome	777	intergenic_lincRNA	2	URS00021CD3A5_29158	3.0.E+00	72
			Contig_0057951_	19	Cytoplasm	803	intergenic_lincRNA	6	URS00021AD0E2_29158	1.3.E+04	61
			Contig_0038930_	16	Nucleolus, Nucleus	789	intergenic_lincRNA	8	URS000219CCA6_29158	3.5.E+03	58
		TCo	Contig_0140862	-679	Ribosome	834	intergenic_lincRNA	88	URS00021B5BD2_29158	4.9.E+00	66
			Contig_0160090	-333	Nucleolus, Nucleus	210	intergenic_lincRNA	1	URS00021C8C52_29158	3.7.E+03	59
			Contig_0071475_	-41	Cytoplasm	826	sense_intronic_ncRNA	3	URS00021E9FB5_29158	1.5.E-73	97
			Contig_0018427_	-31	Nucleolus, Nucleus	798	intergenic_lincRNA	4	URS00021C6183_29158	4.7.E+03	62
			Contig_0159682	-30	Nucleolus, Nucleus	176	sense_intronic_ncRNA	–	–	–	–
			Contig_0110582_	-26	Cytoplasm	816	intergenic_lincRNA	–	–	–	–
			Contig_0056066_	-22	Nucleolus, Nucleus	793	intergenic_lincRNA	7	URS00021D148C_29158	2.6.E-61	92
			Contig_0079327_	-21	Nucleolus, Nucleus	698	antisense_lncRNA	2	URS00021CFE8F_29158	3.4.E+04	60
			Contig_0184303	-20	Cytoplasm	154	sense_intronic_ncRNA	–	–	–	–
			Contig_0029718_	-19	Exosome	826	sense_intronic_ncRNA	7	URS00021AB809_29158	3.9.E+03	63
(B)	TYa vs. AYa	AYa	Contig_0035605_	384	Cytoplasm	783	intergenic_lincRNA	–	–	–	–
			Contig_0012027_	252	Cytoplasm	743	intergenic_lincRNA	7	URS00021C0E1D_29158	8.5.E+03	71
			Contig_0088304_	244	Cytoplasm	750	antisense_lncRNA	4	URS00021B060E_29158	3.7.E+03	85
			Contig_0086064_	228	Cytoplasm	758	antisense_lncRNA	3	URS00021E3218_29158	6.4.E+04	56
			Contig_0016027_	112	Nucleolus, Nucleus	826	antisense_lncRNA	3	URS00021BFCC5_29158	6.8.E+03	66
			Contig_0037768_	88	Exosome	792	antisense_lncRNA	240	URS00021C2727_29158	1.1.E-09	58
			Contig_0011051_	50	Ribosome	826	intergenic_lincRNA	–	–	–	–
			Contig_0043924_	40	Cytoplasm	784	sense_intronic_ncRNA	9	URS00021DA0C6_29158	1.3.E+04	62
			Contig_0005394_	37	Exosome	828	antisense_lncRNA	2	URS00021EAC71_29158	5.2.E+03	63
			Contig_0121672_	30	Cytoplasm	497	sense_intronic_ncRNA	4	URS00021BD644_29158	2.8.E+01	59
		TYa	Contig_0032324_	-502	Cytoplasm	842	intergenic_lincRNA	–	–	–	–
			Contig_0023274_	-61	Ribosome	802	intergenic_lincRNA	5	URS00021B266E_29158	3.8.E+02	57
			Contig_0043683_	-36	Cytoplasm	739	intergenic_lincRNA	15	URS00021B523D_29158	4.1.E+04	67
			Contig_0065844_	-28	Cytoplasm	804	intergenic_lincRNA	3	URS00021A130D_29158	1.2.E+04	48
			Contig_0006814_	-27	Cytoplasm	791	antisense_lncRNA	5	URS00021DA969_29158	3.1.E+03	63
			Contig_0082385_	-25	Cytoplasm	813	antisense_lncRNA	8	URS00021D9920_29158	3.1.E+03	54
			Contig_0100429_	-24	Cytoplasm	795	intergenic_lincRNA	3	URS00021BE419_29158	8.0.E+04	67
			Contig_0011936_	-21	Ribosome	760	sense_intronic_ncRNA	6	URS00021DA80F_29158	1.3.E+04	57
			Contig_0081009_	-17	Cytoplasm	839	sense_intronic_ncRNA	2	URS00021903C8_29158	7.0.E+03	59
			Contig_0158461	-15	Nucleolus, Nucleus	310	antisense_lncRNA	2	URS00021DA27E_29158	3.4.E+03	68

Results of the annotations for top ten up-regulated lncRNAs (DE-lncRNAs) detected in each sample comparison under the home vs. away (δ_HA_) criterion for Cochamó (A) and Yaldad (B). Labels: ACo and TCo, self- and cross- transplanted individuals from Cochamó; respectively. Likewise, AYa and TYa, self- and cross- transplanted individuals from Yaldad; Mga, *Mytilus galloprovincialis*; Fold change values of transplanted individuals are represented as negative numbers.

### 3.7 Annotation of NPC-genes

The genomic mapping of DE-lncRNAs to the *M. chilensis* chromosome complement focused on nuclear-localized DE-lncRNAs (29.5% of total detected DE-lncRNAs), based on their predicted subcellular localization. Nuclear localization has been reported in other systems in association with chromatin proximity and transcription-related contexts (9). In this study, these nuclear DE-lncRNAs were found within 10 kb upstream or downstream of NPC genes, a pattern consistent with genomic proximity between DE-lncRNAs and neighboring protein-coding genes, without implying regulatory interactions.

#### 3.7.1 Local vs. foreign (δ_LF_) criterion comparison of NPC-genes

**Table 4** compares DE-lncRNAs with NPC-genes that have nuclear localization, including their FC values and annotated NPC-genes from various databases. Among the DE-lncRNAs identified in ACo (TYa vs. ACo in **Table 4a**), the first two, Contig_0112889_ and Contig_0114921_, showed high FC values (86.45 and 32.48, respectively) and are linked to NPC-genes with sequence similarity to genes annotated as encoding the epidermal growth factor-like domain (eggNOG accession number 7739.XP_002597498.1) and homeobox protein OTX2-like (eggNOG accession number 8010.XP_010880539.1). The former has been associated with cellular signaling and proliferation, while the latter has been described as a transcription factor. In TYa samples, the DE-lncRNAs Contig_0161780 (FC value = -33.86) and Contig_0172359 (FC value = -32.41) showed high fold change values and were linked with MCH027649.1 and MCH009455.1 NPC-genes. The first NPC-gene showed no detectable sequence similarity with any known gene, while the second has homology with the 50S ribosome-binding GTPase protein (Pfam accession number PF01926.22).

**Table 4:** Annotated NPC-genes detected under local vs. foreign (δ_LF_) criterion comparison.

	Comparison	Samples	DE-lncRNA ID	Fold change	Chromosome	NPC-gene ID	Database	Database ID	Description
(A)	TYa vs ACo	ACo	Contig_0112889_	86.45	Chromosome_3	MCH016077.1	eggNOG	7739.XP_002597498.1	Epidermal growth factor-like domain
			Contig_0114921_	32.48	Chromosome_4	MCH020814.1	eggNOG	8010.XP_010880539.1	Homeobox protein OTX2-like
			Contig_0084357_	20.11	Chromosome_8	MCH031251.1	Pfam	PF00131.19	Metallothionein
			Contig_0113545_	14.18	Chromosome_12	MCH008182.1	BLAST NR	OPL20932.1	hypothetical protein AM593_01491
			Contig_0071957_	13.91	Chromosome_9	MCH033457.1	BLAST NR	EKC31123.1	hypothetical protein CGI_10028754
			Contig_0101395_	12.50	Chromosome_7	MCH027153.1	eggNOG	7739.XP_002608841.1	Glycosyl transferases group 1
		TYa	Contig_0161780	-33.86	Chromosome_7	MCH027649.1	–	–	–
			Contig_0172359	-32.41	Chromosome_12	MCH009455.1		PF01926.22	50S ribosome-binding GTPase
			Contig_0169973	-30.75	Chromosome_7	MCH027619.1	–	–	–
			Contig_0149767	-16.67	Chromosome_13	MCH011040.1	–	–	–
			Contig_0032265_	-16.22	Chromosome_12	MCH009266.1	BLAST NR	ANN45949.1	byssal amine oxidase-like protein 1
			Contig_0131469	-16.19	Chromosome_8	MCH031417.1	Pfam	PF00194.20	Eukaryotic-type carbonic anhydrase
			Contig_0033969_	-13.33	Chromosome_11	MCH007442.1	eggNOG	7739.XP_002594892.1	cell surface recognition signaling
(B)	TCo vs AYa	AYa	Contig_0112788_	250.40	Chromosome_14	MCH011872.1	–	–	–
			Contig_0145936	199.75	Chromosome_11	MCH005590.1	–	–	–
			Contig_0107622_	105.91	Chromosome_11	MCH006631.1	Swissprot	A0A210QSJ3	Chitin synthase
			Contig_0005745_	102.25	Chromosome_3	MCH017892.1	Pfam	PF00057.17	Low-density lipoprotein receptor
			Contig_0053441_	93.24	Chromosome_11	MCH006631.1	Swissprot	A0A210QSJ3	Chitin synthase
			Contig_0016027_	41.82	Chromosome_10	MCH005140.1	–	–	–
			Contig_0011692_	41.75	Chromosome_7	MCH028101.1	Pfam	PF04297.13	Putative helix-turn-helix protein
			Contig_0047019_	25.58	Chromosome_11	MCH005590.1	–	–	–
			Contig_0047483_	24.56	Chromosome_8	MCH030467.1	BLAST NR	XP_022326523.1	uncharacterized protein LOC111126294
			Contig_0043139_	16.08	Chromosome_6	MCH024519.1	eggNOG	7668.SPU_004811-tr	retrotransposable element Tf2–155 kDa
		TCo	Contig_0003123_	-400.71	Chromosome_12	MCH008030.1	Pfam	PF00147.17	Fibrinogen beta and gamma chains
			Contig_0156322	-67.12	Chromosome_13	MCH010870.1	BLAST NR	OPL33888.1	hypothetical protein AM593_03742
			Contig_0087620_	-42.14	Chromosome_6	MCH025983.1	eggNOG	7739.XP_002613072.1	carbohydrate binding
			Contig_0158461	-32.12	Chromosome_13	MCH011453.1	–	–	–
			Contig_0119107_	-19.92	Chromosome_14	MCH013517.1	eggNOG	6500.XP_005111527.1	corticospinal neuron axon guidance
			Contig_0038608_	-15.77	Chromosome_7	MCH028568.1	Swissprot	A0A1S3H5B5	putative nuclease HARBI1
			Contig_0152550	-14.93	Chromosome_5	MCH021917.1	BLAST NR	XP_011444779.1	isochorismatase domain-containing protein
			Contig_0083249_	-10.80	Chromosome_10	MCH004847.1	eggNOG	126957.SMAR007392-PA	regulation of DNA methylation

Annotations using diverse databases (SwissProt, pfam, BLAST NR, eggNOG) of 10 kb up- and down-stream neighboring protein coding genes (NPC-genes) of DE-lncRNAs with nuclear subcellular localization, detected in each sample comparison under the local vs. foreign (δ_LF_) criterion for Cochamó (A) and Yaldad (B). Fold change values of transplanted individuals are presented as negative numbers. Labels: ACo and TCo = self- and cross- transplanted individuals from Cochamó, respectively. Likewise, AYa and TYa = self- and cross- transplanted individuals from Yaldad.

On the other hand, for the TCo vs. AYa comparison (**Table 4b**), the DE-lncRNAs Contig_0107622_ (FC value= 105.91) and Contig_0005745_ (FC value= 102.25) stand out in AYa samples. The NPC gene annotated for the former was chitin synthase (SwissProt accession number A0A210QSJ3), a gene previously associated with shell biomineralization in mussels. The NPC-gene for the latter was a low-density lipoprotein receptor (Pfam accession number PF00057.17), a gene previously associated with cholesterol uptake and lipid metabolism. In TCo samples, the DE-lncRNAs Contig_0003123_ (FC value= -400.71) and Contig_0156322 (FC value= -67.12) were located near MCH008030.1 and MCH010870.1 NPC-genes. The former NPC gene showed sequence similarity with fibrinogen beta and gamma chains (Pfam accession number PF00147.17), while the latter homologs with a hypothetical protein AM593_03742 (NR accession number OPL33888.1).

#### 3.7.2 Home vs. away (δ_HA_) criterion comparison of NPC-genes

**Table 5** lists, for the δ_HA_ criterion in both locations, the DE-lncRNAs with linked NPC-genes and predicted nuclear subcellular localization. From the list of DE-lncRNAs detected in ACo (from TCo vs. ACo comparison in **Table 5a**), the first two (Contig_0115849_ and Contig_0168455) with high FC values (102 and 52.99, respectively) exhibited sequence similarity to genes encoding proteins associated with insulin-like growth factor availability and activity, which have been described in relation to control growth, development, and metabolism in other organisms (NR accession number XP_021351702.1), and caspase domain, which have been associated with programmed cell death and inflammation responses (Pfam accession number PF00656.21). In TCo samples, the DE-lncRNAs Contig_0160090 (FC value = -332.88) and Contig_0018427 (FC value = -31.25) and were linked with NPC-genes MCH008259.1 and MCH019830.1. The former showed sequence similarity to a zinc ion-binding protein (eggNOG accession number 6500.XP_005093449.1), while the latter showed similarity to a gene involved in regulating replicative cell aging (eggNOG accession number 10224.XP_006817652.1).

**Table 5:** Annotated NPC-genes detected under home vs. away (δ_HA_) criterion comparison.

	Comparison	Samples	DE-lncRNA ID	Fold change	Chromosome	NPC-gene ID	Database	Database ID	Description
(A)	TCo vs. ACo	ACo	Contig_0115849_	102.00	Chromosome_5	MCH024276.1	BLAST NR	XP_021351702.1	insulin-like growth factor-binding complex
			Contig_0168455	52.99	Chromosome_8	MCH031744.1	Pfam	PF00656.21	Caspase domain
			Contig_0069732_	30.66	Chromosome_13	MCH011095.1	Pfam	PF07714.16	Protein tyrosine kinase
			Contig_0026577_	22.17	Chromosome_1	MCH001721.1	Pfam	PF13837.5	Myb/SANT-like DNA-binding domain
			Contig_0038930_	16.12	Chromosome_5	MCH023695.1	BLAST NR	OWF42392.1	hypothetical protein KP79_PYT05157
			Contig_0029680_	12.85	Chromosome_2	MCH013755.1	–	–	–
			Contig_0112889_	10.22	Chromosome_3	MCH016077.1	eggNOG	7739.XP_002597498.1	Epidermal growth factor-like domain
		TCo	Contig_0160090	-332.88	Chromosome_12	MCH008259.1	eggNOG	6500.XP_005093449.1	zinc ion binding
			Contig_0018427_	-31.25	Chromosome_4	MCH019830.1	eggNOG	10224.XP_006817652.1	regulation of replicative cell aging
			Contig_0159682	-30.34	Chromosome_3	MCH016646.1	BLAST NR	XP_013393141.1	uncharacterized protein LOC106160914
			Contig_0056066_	-21.68	Chromosome_7	MCH028599.1	BLAST NR	XP_022309634.1	uncharacterized protein LOC111115259
			Contig_0079327_	-20.60	Chromosome_13	MCH010610.1	Pfam	PF13582.5	Metallo-peptidase family M12B Reprolysin
			Contig_0126635_	-14.86	Chromosome_11	MCH005770.1	Swissprot	K1RDD5	SCF E3 ubiquitin ligase complex F-box
			Contig_0021314_	-11.24	Chromosome_1	MCH002487.1	Pfam	PF00091.24	Tubulin, constituent of cytoskeleton
(B)	TYa vs. AYa	AYa	Contig_0016027_	112.03	Chromosome_10	MCH005140.1	–	–	–
			Contig_0158874	27.57	Chromosome_8	MCH029901.1	eggNOG	345341.KUTG_08091	Methyltransferase domain
			Contig_0114779_	18.50	Chromosome_11	MCH007697.1	–	–	–
			Contig_0047483_	13.29	Chromosome_8	MCH030467.1	BLAST NR	XP_022326523.1	uncharacterized protein LOC111126294
		TYa	Contig_0158461	-14.76	Chromosome_13	MCH011453.1	–	–	–
			Contig_0149793	-13.87	Chromosome_13	MCH011394.1	eggNOG	7739.XP_002594723.1	C-type lectin or carbohydrate-recognition
			Contig_0043746_	-13.58	Chromosome_10	MCH004063.1	Pfam	PF00106.24	short chain dehydrogenase
			Contig_0127652_	-12.65	Chromosome_8	MCH031101.1	–	–	–

Annotations using diverse databases (SwissProt, pfam, BLAST NR, eggNOG) of 10 kb up- and down-stream neighboring protein coding genes (NPC-genes) of DE-lncRNAs with nuclear subcellular localization, detected in each sample comparison under the home vs. away (δ_HA_) criterion for Cochamó (A) and Yaldad (B). Fold change values of transplanted individuals are presented as negative numbers. Labels: ACo and TCo, self- and cross- transplanted individuals from Cochamó, respectively; Likewise, AYa and TYa, self- and cross- transplanted individuals from Yaldad.

On the other hand, in the TYa vs. AYa comparison (**Table 5b**), the DE-lncRNAs Contig_0016027_ (FC value = 112.03) and Contig_0158874 (FC value = 27.57) showed high fold change values in AYa samples. While the NPC-gene annotated for the former showed no detectable sequence similarity, the latter showed similarity with a methyltransferase domain, a protein previously described as involved in DNA methylation processes (eggNOG accession number 345341.KUTG_08091). In TYa samples, the DE-lncRNAs Contig_0158461 (FC value = -14.76) and Contig_0149793 (FC value = -13.87) were identified and linked with MCH011453.1 and MCH011394.1 NPC-genes. The NPC-gene annotated for the former showed no detectable sequence similarity, whereas the latter showed sequence similarity to a C-type lectin recognition domain, which has been associated with pathogen recognition and immune adhesion (eggNOG accession number 7739.XP_002594723.1).

### 3.8 NPC-genes vs. caDEGs expression comparison

**Figure 5:**
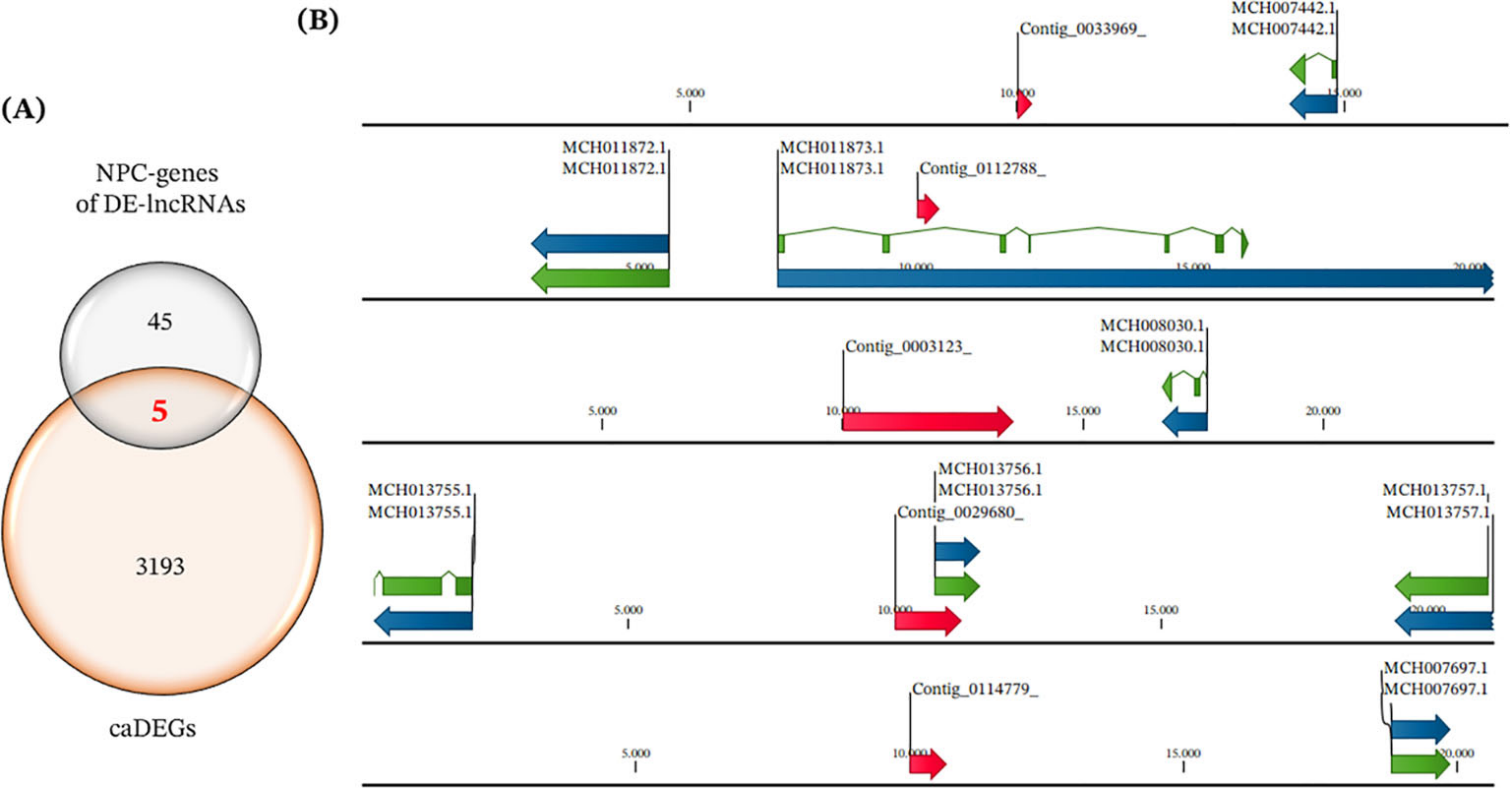
Overlap between DE-lncRNAs-linked NPC-genes and candidate adaptive DEGs (caDEGs). **(A)** Venn diagram showing the intersection between NPC-genes of DE-lncRNAs (n = 50) and caDEGs (n = 3198) reported by Yévenes et al. (2025) (49). Only five genes were shared between the two sets, representing caDEGs and NPC-genes. **(B)** Genomic maps of the five caDEGs-NPC-genes in the Mytilus chilensis genome sequence. Arrows represent annotated protein-coding genes in DNA and mRNA (blue and green, respectively) and DE-lncRNAs (red), with their orientation indicated. Distances between loci are given in kilobases.

The comparison between the set of neighboring protein-coding genes (NPC-genes) of the DE-lncRNAs identified in this study and the candidate adaptive differentially expressed genes (caDEGs) reported (49) showed limited overlap (**Figure 5A**). Out of the 50 NPC-genes linked with DE-lncRNAs, only five overlapped with the caDEGs, while 45 and 3193 were exclusive to each set. These five shared genes were annotated (**Table 6**), mapped to different chromosomes in the *M. chilensis* genome sequence, and were located in close genomic proximity to DE-lncRNAs (**Figure 5A**). Notably, negative fold-change values reflected transcriptional variation in transplanted individuals, while positive values corresponded to self-transplanted ones. Functional annotation revealed that one of these genes encodes a protein involved in cell surface recognition signaling (MCH007442.1, eggNOG database), and another showed sequence similarity to fibrinogen beta and gamma chains (MCH008030.1, Pfam database). The remaining genes are uncharacterized. This co-localization is consistent with a close genomic association between DE-lncRNAs and caDEGs, although it does not by itself demonstrate cis-regulatory relationships.

**Table 6:** Fold change comparison between NPC-genes and caDETs.

Comparison	DE-lncRNA ID	Sample	Fold change DE-lncRNAs	NPC-gene	Sample	Fold change NPC-gene	Database	Database ID	Description
TYa vs ACo	Contig_0033969_	TCo	-13.33	MCH007442.1	TCo	-3.07	eggNOG	7739.XP_002594892.1	cell surface recognition signaling
TCo vs AYa	Contig_0112788_	AYa	250.40	MCH011872.1	AYa	5.16	–	–	–
TCo vs AYa	Contig_0003123_	TYa	-400.71	MCH008030.1	AYa	3.82	Pfam	PF00147.17	fibrinogen beta and gamma chains
TCo vs ACo	Contig_0029680_	ACo	12.85	MCH013755.1	TCo	-3.77	–	–	–
TYa vs AYa	Contig_0114779_	AYa	18.50	MCH007697.1	AYa	35.09	–	–	–

Comparison of the Fold change (FC) values between neighboring DE-lncRNA protein coding genes (NPC-genes) and candidate adaptive differentially expressed genes (caDEGs), detected in each sample comparison under local vs. foreign (δ_LF_) and home vs. away (δ_HA_) criteria. Fold change values of transplanted individuals are presented as negative numbers. Labels: ACo and TCo, self- and cross- transplanted from Cochamó, respectively; Likewise, AYa and TYa, self- and cross- transplanted from Yaldad.

## 4 Discussion

This study expands the framework of local adaptation in *Mytilus chilensis* by extending previous genomic evidence (46–49) to a non-coding regulatory epigenomic layer. Those studies showed that, despite high larval connectivity (58–60) and aquaculture-related translocations in a system showing signs of panmixia and low large-scale genetic structure (61), natural seedbeds in Cochamó and Yaldad exhibit patterns consistent with local adaptation in genome functioning, linked to growth, immunity, osmoregulation, and metabolism, supporting the interpretation that local selective pressures may counteract the homogenizing effect of gene flow.

In reciprocal transplant experiments, the contrasts local versus foreign (δ_LF_) and home versus away (δ_HA_) capture key dimensions relevant to local adaptation. While δ_LF_ indicates how well local genotypes compete against foreign ones within each habitat, δ_HA_ quantifies the cost/benefit of moving away from the native environment for each population, reflecting differences in their ecological performance. Therefore, the combined interpretation of δ_LF_ (>0) and δ_HA_ (>0) provides a useful framework to interpret patterns consistent with local adaptive processes (63, 64). Likewise, asymmetries between these parameters reveal environmental heterogeneity and differences in population sensitivity. In this study, lncRNA expression profiles exhibited patterns consistent with local adaptive signals under both criteria. The heatmap clustering revealed that expression profiles are primarily structured by population of origin, reflecting baseline differences between Cochamó and Yaldad. However, additional sub-structuring within each location indicates that transplantation also contributes to expression variability, although to a lesser extent than location-driven effects. In addition, transplanted individuals up-regulated a larger number of differentially expressed lncRNAs (DE-lncRNAs) and exhibited greater fold change than their self-transplanted counterparts (**Figure 3**). This asymmetry is consistent with a possible cost of exposure to non-native environments and aligns with patterns previously described for differentially expressed adaptive candidate genes (caDEGs) and mapped outlier SNPs in the same individuals (49). Thus, the convergence of patterns across molecular levels (genomic and epigenomic) suggests that, despite their distinctive molecular natures, both exhibit convergent patterns consistent with adaptive responses in this species, in line with the low genetic divergence reported and with patterns previously interpreted as adaptive signal concentrated in candidate loci (52, 62).

While alternative explanations such as stress responses, tissue-specific effects, or the temporal scale of the experiment may contribute to the observed patterns, these like reflect interacting components of genotype–environment responses rather than a single underlying mechanism. Accordingly, the transcriptomic patterns observed under transplant conditions may reflect a combination of plastic and potentially adaptive responses, consistent with local adaptive divergence while acknowledging multiple underlying processes. Overall, the number of DE-lncRNAs and their fold changes were higher in Yaldad, consistent with greater sensitivity to habitat change, whereas mussels from Cochamó maintained relatively stable expression profiles under non-native conditions. These differences align with the contrasting environmental regimes of both locations (**Figure 1**), where Yaldad is characterized by more stable oceanic conditions and Cochamó by greater variability associated with estuarine influence (54–56), with mussels from Yaldad exhibited more pronounced lncRNAs expression shifts when transplanted, whereas those from Cochamó showed less pronounced responses. While not directly demonstrating local adaptation, these patterns are consistent with differential epigenetic responses shaped by environmental context. In metapopulations with high gene flow, epigenetic differentiation has been proposed as a potential mechanism for rapid phenotypic adjustment (69), providing a conceptual framework for interpreting these patterns.

During the mapping of DE-lncRNAs to the genome to identify neighboring protein-coding genes (NPC-genes, 10 kb up- and downstream), a progressive drop in number was observed from the total number to the subset of them linked to NPC-genes and showing extreme expression changes under both δ_LF_ and δ_HA_. The comparative overlap (**Figure 5**) between NPC-genes and previously reported caDEGs was low (5 shared genes out of 3198). However, the co-localization of these caDEGs and DE-lncRNAs may be informative but is not sufficient to infer cis-regulation, hence is consistent with genomic proximity patterns that may facilitate potential cis-associated interactions with caDEGs, which could be interpreted as a preliminary indicator of potential local interactions associated to adaptive processes. This finding also is consistent with cis-associated patterns frequently reported under environmental stress, also described in mollusks and other taxa, in which lncRNAs have been described as local enhancers or modulators of transcription (30, 57). Hence, the low overlap of NPC-genes with caDEGs does not invalidate their relevance; instead, it may reflect associations between DE-lncRNAs and metabolic pathways and adaptive phenotypes through mechanisms acting along different pathways and non-redundant regulatory axes (e.g., chromatin topology or enhancer-like activity) (9, 70, 71), operating across distinct temporal/spatial scales (15), and in ways that complement expression changes in the coding genomic layer represented by caDEGs.

Rather than indicating a weak relationship between coding and non-coding regulatory layers, the limited overlap observed between NPC-genes and caDEGs (five shared genes) may reflect the operation of partially independent but complementary regulatory mechanisms. Long non-coding RNAs have been reported to act through diverse modes of action, including cis- and trans-regulatory effects, chromatin organization, and modulation of transcriptional dynamics, which may not necessarily involve the same set of protein-coding genes identified through differential expression analyses. Additionally, lncRNAs and coding genes may contribute to adaptive responses across different temporal and spatial scales, with lncRNAs potentially acting as fine-tuning or context-dependent regulators, while coding genes reflect more stable expression shifts. At the same time, this pattern may also be influenced by methodological constraints, including incomplete functional annotation, limitations in genome mapping in non-model species, and the inherent difficulty of capturing regulatory interactions based solely on genomic proximity. Taken together, these considerations suggest that this pattern is consistent with a multi-layered regulatory architecture, where coding and non-coding elements contribute in a complementary and non-redundant manner to genotype–environment responses.

The predicted subcellular distribution for the DE-lncRNAs linked to NPC-genes supports this interpretation. While these results do not directly demonstrate regulatory mechanisms, they are consistent with patterns reported in other organisms (9, 71). Approximately 70% of the DE-lncRNAs were predicted to be localized in cytoplasmic, ribosomal, and exosomal compartments, while only about 30% were predicted to nuclear localization. As cytoplasmic DE-lncRNAs have been proposed as rapid-response regulatory elements associated with post-transcriptional processes, including translation, mRNA stability, and cell signaling. The annotated ribosome-associated lncRNAs may play a role in modulating protein synthesis under stress, such as transplantation. Also, the annotated exosomal DE-lncRNAs may play a role in intercellular communication, potentially contributing to coordinated adaptive responses at the tissue level.

Methodologically, in silico annotation of lncRNAs has known limitations when attempting to link them to a biological function. Accordingly, quality was prioritized over data quantity, and standardization/benchmarking practices of predictors (e.g., subcellular localization) were adopted to strengthen the functional inferences (72). Thus, iLoc-LncRNA and RNACentral were integrated to triangulate localization and sequence similarity; BLAST alignments showed 48-73% identity between DE-lncRNAs and *Mytilus galloprovincialis* sequences, with some high-identity exceptions (up to 97%). Although these alignments indicate sequence similarity, by themselves they are insufficient to assign function, since the effects of many lncRNAs depend on locus context and subcellular localization. Hence, the need for complementary experimental approaches to better characterize the potential functional roles of DE-lncRNAs detected in *M. chilensis*; for example, CRISPRi or focal deletions in lncRNA promoters/enhancers without altering the NPC-gene; ChIRP/CHART and conformation capture (3D/HiChIP) to test lncRNA–chromatin contacts; subcellular fractionation and RNA-FISH to verify localization; and antisense oligonucleotides (ASOs) to assess their influence on NPC-genes and stress-related phenotypes. In addition, multi-tissue assays and environmental gradients (34, 57), together with multigenerational common garden experiments and reciprocal crosses, would help partition genetic, epigenetic, and environmental contributions to adaptive phenotypes and test the hypothesis of epigenetic differentiation in high-gene-flow systems (69). These approaches align with modern strategies for moving from associations toward mechanistic understanding of biological function (9, 73).

While the use of pooled RNA samples prevents the direct estimation of inter-individual variability and may underestimate biological dispersion, this design should be interpreted within the genomic context of mussels. In these organisms, high levels of gene presence/absence variation (PAV) have been reported, with up to ~38% of genes being dispensable in *Mytilus galloprovincialis* (74), resulting in substantial differences in gene content among individuals. Under this framework, transcriptomic variability may reflect not only regulatory differences but also underlying gene presence or absence. Consequently, pooling multiple individuals may contribute to capturing a broader gene repertoire and a more representative fraction of population-level genetic diversity, partially reducing biases associated with individual-specific gene content, thereby providing a more integrative view of population-level transcriptional patterns. Therefore, although our results are not intended to resolve intra-population variability, they provide an integrative perspective on transcriptional responses in a system characterized by extensive genomic heterogeneity.

The large number of transcripts evaluated in this study also introduces an inherent risk of false positives, even when applying FDR-based correction. In this context, the combined use of statistical significance and stringent fold change thresholds can be interpreted as a conservative strategy to prioritize robust meaningful signals. While more restrictive approaches, such as Bonferroni correction or extremely high fold change thresholds, which resulted in the complete loss of detectable differentially expressed lncRNAs, suggesting an increased risk of false negatives. Therefore, this framework reflects a balance between sensitivity and specificity, with an emphasis on identifying strong and consistent expression patterns rather than maximizing the detection of marginal signals. Under this perspective, the results should be interpreted as hypothesis-generating, emphasizing the identification of candidate lncRNAs for future validation rather than providing an exhaustive catalog of differential expression. Further studies using complementary analytical frameworks may help refine the identification of DE-lncRNAs and further explore the patterns observed. While CLC Genomics Workbench provides a GLM-based framework, alternative tools such as DESeq2 or edgeR offer more explicitly documented approaches for dispersion estimation and shrinkage, which could be explored in future studies.

In mechanistic terms, the evidence points to lncRNA loci as functionally relevant genomic elements as they are associated with chromatin organization, enhancer-like activity, and the nuclear formation of transcriptional condensates; their behavior depends on both locus context and subcellular localization (9, 10, 57). Moreover, part of the regulatory signal may reside in local DNA architecture or in the act of transcription itself (boundary/enhancer effect) rather than (and not necessarily) in the RNA molecule (11). Altogether, this is consistent with an interpretation of the complementary, non-redundant genomic and epigenomic regulatory layers. In this context, the results of this study may have important implications for the conservation and management of the natural *M. chilensis* beds that serve as seed sources for aquaculture in southern Chile. The patterns observed for DE-lncRNAs, consistent with regulatory genomic processes associated with local adaptation, highlight the importance of protecting the biological diversity of each natural bed, given their role as seed sources for the industry and their documented vulnerability (47, 51, 52), potentially helping to prevent genetic and epigenetic homogenization that could weaken the species’ resilience to environmental changes and climate challenges.

## 5 Conclusion

This study highlights the importance of incorporating epigenetic mechanisms into the conservation and management of natural *Mytilus chilensis* beds used in aquaculture. Although the linkage between DE-lncRNAs and previously identified candidate adaptive genes (caDEGs) was limited and functional validation is still required, our results support the interpretation that lncRNAs may represent a complementary and dynamic regulatory layer associated with patterns consistent with local adaptation. In this context, these associations primarily reflect genomic proximity and do not necessarily imply regulatory relationships, they remain informative for identifying spatial patterns of gene organization and potential regulatory contexts relevant to adaptive processes. Furthermore, these findings open new avenues for understanding the evolution of this species, suggesting that epigenetic regulation via lncRNAs may represent one of the mechanisms contributing to rapid responses to environmental change without relying solely on changes in DNA sequence. These findings provide a more nuanced and comprehensive view of the evolutionary processes that allow species with high gene flow, like *M. chilensis*, to persist and thrive in heterogeneous environments. Future research focused on experimental validation and assessing the heritability of these epigenetic signals will be essential to better understand their contribution to the long-term resilience, adaptive potential, and the evolutionary trajectory of this species in a changing environment.

## Data Availability

The datasets presented in this study can be found in online repositories. The names of the repository/repositories and accession number(s) can be found in the article/supplementary material.
